# Topographic measurements of eyelids and orbit in enucleated eyes with hydroxyapatite integrated implant versus PMMA implant

**Published:** 2014

**Authors:** S Gradinaru, M Totir, R Iancu, C Leasu, S Pricopie, S Yasin, R Ciuluvica, E Ungureanu

**Affiliations:** * UMF “Carol Davila” Ophthalmology Department, 8 Eroilor Sanitari Blvd, Bucharest, Romania; ** UMF “Carol Davila” Anatomy Department, 8 Eroilor Sanitari Blvd, Bucharest, Romania; *** University Emergency Hospital Bucharest, 126 Splaiul Independentei Blvd, Bucharest, Romania

**Keywords:** hydroxyapatite, horizontal eyelid fissure, eyelid fissure height

## Abstract

**Introduction: **This study reports our results relating to palpebral eyelid fissure and orbital measurements following evisceration with orbital implantation of hydroxyapatite integrated implant and PMMA implant.

**Materials and Methods:** This study is a prospective study of 43 patients that underwent evisceration for different ocular affections at University Emergency Hospital Bucharest, Ophthalmology department between January 2009 and September 2010 (Group A comprising of twenty patients had the coralline hydroxyapatite implant –Integrated Ocular Implants, USA and Group B comprising of twenty-three received non-integrated PMMA ocular implants) .The outcomes measured were the degree of exo /enophthalmos, horizontal eyelid fissure and palpebral fissure height at 4 years after surgical intervention related to measurement to the contralateral eye.

**Results:** Horizontal eyelid fissure (HEF) was suffering a shortening of 7.4% in the group B versus the contralateral eye, and only 1.9% in the group A related to the contralateral eye.

Eyelid fissure height was greater in the group B with 5.2% regarding the contralateral eye, and 1.2% in group A.

The degree of enophthalmia was higher in the group B of 4 mm versus the contralateral eye and lower in group A 1.5 mm regarding the contralateral eye.

**Conclusions:**. Although a hydroxyapatite implant may be not as economic as a PMMA implant, a patient must be warned about the effect on its ocular structures in time and that cosmetic appearance over years will change more dramatically than in the fellow normal eye. Therefore preoperative counseling of the patient is crucial in long term patient satisfaction.

## Introduction

The orbit is a conical or four-sided pyramidal cavity, symmetrical situated, with a rectangular base situated anteriorly and its apex situated postero-medially. The base which opens in the face has four borders. The floor is formed by the orbital surface of maxilla, the orbital surface of zygomatic bone and the orbital process of palatine bone. The medial wall is formed by the orbital plate of the ethmoid bone, as well as contributions from the frontal process of maxilla, the lacrimal bone and a small part of the body of the sphenoid. The lateral wall is formed by the frontal process of zygomatic and more posteriorly by the orbital plate of the greater wing of sphenoid. In this pyramidal space, with a volume of about 30 cm3, lies orbital fat surrounding the globe, extraocular muscles, nerves and a vascular bundle, the orbital fat acting like a cushion for globe protection and surrounding structures. [**1**] Eyelids are musculocutaneous structures that also have a protective role for the globe. The upper and lower lid are joint at medial and lateral region, delimitating a space –eyelid fissure with specific measurements regarding sex and ageș (see **Table.1**).

**Table 1:** Normal orbital and palpebral parameters

Volume	30 cm³
Orbital aperture width	40 mm
Orbital aperture length	35 mm
Horizontal eyelid fissure women	26.7mm
Horizontal eyelid fissure men	27.2 mm
Exophthalmometry women	18 mm
Exophthalmometry men	20 mm
Palpebral fissure height women	10-12 mm
Palpebral fissure height men	9-10 mm

The upper lid is larger and more mobile than the lower lid (due to levator palpebrae muscle) and in primary gaze position the upper lid covers about 2mm of the superior margin of cornea, while the lower lid margin is at the inferior sclero-corneal limbus. Structurally the lid has from inside out conjunctiva, muscular fibers, tarsus and orbitary septum, orbicularis muscle and levator palpebrae muscle, subcutaneous tissue and palpebral skin. [**1**]

Aging may cause laxity of eyelid tissues and atrophy of the orbital fat. These changes contribute to the aetiology of several eyelid disorders such as ectropion, entropion, dermatochalasis, and aponeurogenic blepharoptosis. Such aging changes may also affect the position of the eyelids, eyeball, and eyebrow. Moreover, prior surgical intervention of these structures may contribute more to the final cosmetic appearance after evisceration and implantation of an orbital implant. [**2**] Removal of an eye following enucleation or evisceration [**3**,**4**,**5**] creates an orbital soft tissue volume deficiency [**6**], which if it’s not corrected with a proper implant results in a post-enucleation socket syndrome described by Tyers and Collin (with abnormally deep superior sulcus, enophthalmic appearance, upper eyelid ptosis and lower lid malposition). [**6**] Proper implant volume can be established after assessing the axial length by a B scan( ultrasound) preoperatively.

PMMA implants were used many years before porous integrated orbital implants and they are preferred because of low costs, but the hydroxyapatite implant provides a series of advantages: is easily accepted by the organism due to the similarity in structure with human bone tissue, the risk of migration of the implant is reduced due to vascular proliferation inside the ball and introducing the peg permits better movement of the ocular prosthesis. The implant has a number of disadvantages: the costs of the implant and the second stage drilling procedure [**7**].

## Materials and methods

This study’s purpose is to evaluate topographic measurements of eyelids (horizontal eyelid fissure, eyelid fissure height) and orbit (exophthalmometry) of the hydroxyapatite ocular implant compared to non-integrated (PMMA) ocular implants in eviscerated patient at University Emergency Hospital Bucharest from January 2009 to September 2010. 

We had two groups of patients that were age and sex matched: group A (11 women and 9 men) that underwent evisceration with hydroxyapatite implant and group B (13 women and 10 men) that underwent evisceration with PMMA implant, with ages between 45-60 years. One surgeon performed evisceration surgery followed by implantation of a hydroxyapatite ocular implant or PMMA implant, and all topographic measurements were made by three different doctors, four years after the initial surgical procedure.

The patients case reports and intraoperative reports were retrieved for recording the type of implant, implant and socket complications.

Evisceration was proposed as end stage procedure after obtaining the informed consent from the patient and the main indications were: painfully blind eye (neovascular glaucoma in 15 patients, 2 patients with endophthamitis) and ocular trauma (26 patients).

Exclusion criteria were previous lid surgery, previous orbital surgery and orbital or lid trauma. Patients that developed complications in this time were excluded.

In this study only patients that underwent the same standard surgical procedure for evisceration and ocular implantation were considered. The anesthetic procedure for non-integrated ocular implant was the same for all patients consisting of local retrobulbar anesthesia. The surgical technique consisted in 360 degree corneal limbus peritomy, followed by extraction of the iris, lens, choroid, vitreous and retina was performed using a curette and the cauterization of ophthalmic artery performed. The non-integrated ocular implant (PMMA) was placed, followed by the suture of scleral sac, Tenon’s and conjunctiva. A plastic conformer was placed; antibiotics and anti-inflammatory drugs were given for 5 days. After 6 weeks the patients were referred for prosthesis fitting.

The procedure for hydroxyapatite ocular implant was the same in all patients and consisted of the same steps as for non-integrated orbital implants. The hydroxyapatite implant’s size was in all cases decided after performing an A and B scan (Sonomed, EZ AB5500+ A-Scan/B-Scan) of the globe for axial measurement and was in all cases used Bio-eye hydroxyapatite implant (Integrated Ocular Implants-USA). After the placement of the implant in the scleral sac, four posterior sclerectomies were performed in order to obtain fibrovascular ingrowth. The scleral sac, Tenon’s and conjunctiva were sutured. All patients had conformers placed; antibiotics and anti-inflammatory drugs were given for 5 days. After 6 weeks the patients were referred for prosthesis fitting. Approx. 6 months after surgery the peg was placed under local anesthesia, the technical procedure being the same for all patients with integrated orbital implants.

All patients with integrated orbital implant performed a CT scan with contrast substance for the assessment of vascularization inside the implant. A month after the peg placement the final prosthesis was adapted. The final prosthesis in all cases (integrated and nonintegrated implanted patients) was made by the same technician.

All patients were observed in these 4 years but only the outcomes in the 4th year post surgery were considered.

Topographic measurements were made with a compass for horizontal lid fissure and eyelid fissure height and the degree of exopthalmos/enophthalmos was assesed with Hertel exophthalmometer. The size of the horizontal eyelid fissure (HEF) was expressed as the distance between the most nasal point of the medial canthus and the intersection of the vertical line through the lateral canthus with the reference line.

## Results

The main indication for evisceration was painful blind eye, and the second indication was ocular trauma. All patients received implants at the moment of surgery as first choice. Twenty patients received hydroxyapatite integrated ocular implant and twenty-three received non-integrated ocular implants (PMMA).

The patients were monitored for four years but the most significant outcomes were registered after this period compared to the fist year’s measurements; in the hydroxyapatite integrated implant (group A) the most significant variation of the elements was observed after 24 months postoperatively and in the group B the most significant variation was observed after 4 years postoperatively.

There was no statistical difference between the two groups regarding age and sex. (p>0.05)

Horizontal eyelid fissure (HEF) was suffering a shortening of 7.4% in the group B versus the contralateral eye, and only 1.9% in the group A related to the contralateral eye.

Eyelid fissure height was greater in the group B with 5.2% regarding the contralateral eye, and 1.2% in group A.

The degree of enophthalmia was higher in the group B of 4 mm versus the contralateral eye and lower in group A 1.5 mm regarding the contralateral eye.

## Discussion

Aging primarily affects the size of the horizontal eyelid fissure. Van den Bosch [**2**] and al showed a normal reduction of HEF after 45 years of age with more than 10% (in 15 years) and this shortening from the age of 45 years onwards is likely to be due to progressive laxity of the medial and lateral canthal structures. The laxity is more evident in the group B where the movement of the prosthesis is minimal, whereas in group A where the prosthesis movement was similar to the contralateral eye the laxity seems to reach the levels for the age.[**8**]

The atrophy of the orbital fat is responsible for the involutional enophthalmos, but this is larger in the group B so our hypothesis is that grossly underestimated orbital volume loss and inadequate volume replacement [**9**] or contracture of the remaining soft tissues, with an equivalent effect as inadequate volume replacement [**2**] are responsible for enophthalmia where the results in group A sustain the hypothesis that disturbance in the normal spatial architecture and tissue relationships of the orbit [**10**] due to insufficient prosthesis motility are responsible for early development of enophthalmos in group B.[**11**]

Our future purpose is to discover a pattern for the orbital fat loss that can be quantified and converted in orbital and palpebral esthetic images in order to be presented to the patients as possible outcomes after many years postoperatively, so the preoperatory decision to be an assumed one regarding the choice of ocular implant.

## Conclusions

Proper preoperative assessment of the eyes and periocular region of both eyes is essential to optimize the surgical outcome and that outcome in time can impact the patient's appearance as aging process may have more. Although a hydroxyapatite implant may be not as economic as a PMMA implant, a patient must be warned about the effect on its ocular structures in time and that cosmetic appearance over years will change more dramatically than in the fellow normal eye.

Future data regarding the degree of orbital fat loss with respect of age in eviscerated patients with various implants may be a scaffold for development of esthetic images for the patients as possible outcomes years after the initial evisceration procedure.
